# Association between adolescent pregnancy and infant mortality: a population-based study

**DOI:** 10.3389/fped.2025.1459594

**Published:** 2025-04-11

**Authors:** Farhana Rahman, Mamunur Rashid, Mohammad Rocky Khan Chowdhury, Manzur Kader

**Affiliations:** ^1^ Department of Public Health Sciences, Faculty of Medicine, University of Lund, Lund, Sweden; ^2^ Unit of Public Health Science, Faculty of Health and Occupational Studies, University of Gävle, Gävle, Sweden; ^3^ Department of Epidemiology and Preventive Medicine, School of Public Health and Preventive Medicine, Faculty of Medicine, Nursing and Health Sciences, Monash University, Melbourne, VIC, Australia; ^4^ Department of Public Health, First Capital University of Bangladesh, Chuadanga, Bangladesh; ^5^ Department of Medical Science, School of Health and Welfare, Dalarna University, Falun, Sweden

**Keywords:** child death, maternal age, teenage pregnancy, young mother, Asia

## Abstract

**Background:**

Bangladesh continues to grapple with a persistently high infant mortality rate, currently at 38 deaths per 1,000 live births. Adolescent pregnancy poses significant health risks for both mothers and infants globally, yet its specific impact on infant mortality in Bangladesh remains underexplored. Therefore, this study aims to investigate the association between adolescent pregnancy and infant mortality in Bangladesh.

**Methods:**

This cross-sectional study analyzed data from the Bangladesh Demographic and Health Survey 2017–18, focusing on 8,759 infants born to women aged 15–49 years. Adolescent pregnancies were categorized into four groups: <16 years, 16–17 years, 18–19 years, and >19 years. Potential covariates included sociodemographic factors (mothers' age, fathers' occupation, religion), contextual factors (place of delivery, access to media and technology), and healthcare utilization (antenatal and postnatal care). Bivariate logistic regression assessed associations between adolescent pregnancy and infant mortality, presenting adjusted odds ratios (AOR) with 95% confidence intervals (CI) while controlling for these covariates.

**Results:**

The mean age of mothers at first birth was 18.53 years. Among the 8,759 infants studied, 328 (3.74%) died before reaching 12 months of age. Infants born to mothers younger than 16 years initially showed higher odds of mortality (AOR: 1.45, 95% CI: 1.05–2.01); this association persisted even after adjusting for sociodemographic and contextual factors (AOR: 1.41, 95% CI: 1.01–1.96). However, after controlling for healthcare utilization, the relationship was no longer statistically significant (AOR: 1.06, 95% CI: 0.56–1.99).

**Conclusions:**

Delaying childbirth from adolescence to adulthood may reduce infant mortality in Bangladesh. However, adolescent pregnancy alone does not increase infant mortality risk after accounting for healthcare utilization, such as antenatal and postnatal care. Improving access to quality healthcare is crucial for lowering infant mortality. Future cohort studies are needed to better understand the relationship between maternal age and infant health outcomes.

## Introduction

Infant mortality is one of the leading public health problems globally, particularly in low- and middle-income countries. Despite substantial progress in reducing infant mortality over the past two decades, it is still a staggering number in low- and medium-income countries. According to the World Health Organization (WHO), approximately 17 million infants die annually, two-thirds within the first month of life, mostly in low- and middle-income countries (1). Infant mortality is the number of deaths of infants under one year per 1,000 live births in a given population or region (2). According to data in 2020, the global infant mortality rate (between birth and 11 months) is 29 per 1,000 live births (2).

In South Asia, infant mortality has declined, but the infant mortality rate has yet been remarkably high at 56 per 1,000 births in Pakistan, 45 in Afghanistan, and 27 in India (3). Bangladesh has reported an infant mortality rate of 38 deaths per 1,000 live births (4), compared to a global rate of 29 (2). The leading causes of infant mortality in Bangladesh are prematurity (29.7%), birth asphyxia and trauma (22.9%), and sepsis (19.9%) (5, 6). Institutional delivery, antenatal care, and high-income households are linked to lower infant mortality rates in Bangladesh (5–8). Maternal chronic health conditions, poor nutrition, and inadequate prenatal care are also associated with infant mortality (9). Moreover, the mother's educational level, age at birth, the weight of the newborn, sex of the child at birth, and place of birth are other factors that affect infant mortality in Bangladesh (5, 7, 8, 10).

In Bangladesh, approximately one-third of adolescent mothers still give birth at home, resulting in a higher risk of neonatal mortality (4), especially among those under 18 years old compared to older mothers (11). Research has shown that giving birth in a healthcare facility can reduce the risk of neonatal mortality and improve neonatal outcomes for mothers in Bangladesh (6). It has been demonstrated that facility-based deliveries can significantly reduce the risk of neonatal mortality, especially for adolescents with a higher rate of newborn mortality. Inadequate prenatal and postnatal care accounts for a substantial proportion of newborn deaths in Bangladesh, highlighting the need to improve maternal healthcare utilization and remove societal and cultural barriers to accessing healthcare services (5, 12, 13). In addition, sociodemographic factors such as education, place of residence, occupation, wealth index, and religion play a pivotal role in adolescents becoming pregnant, which may, in turn, lead to infant mortality (14, 15). A comprehensive review of infant mortality across 24 developin data, confirmed that proper Antenatal Care (ANC) and Postnatal Care (PNC) significantly reduce the risk of infant mortality (16).

Pregnancy during adolescence presents significant risks to both mother and infant. Adolescent mothers face higher rates of complications like preterm birth, low birth weight, and hypertensive disorders, increasing infant mortality risk. Contributing mechanisms include biological immaturity, affecting fetal growth, and socio-economic factors, such as limited access to prenatal care and poor nutrition (17). Children of younger mothers face greater disadvantages, with maternal immaturity playing a critical role (18). Additionally, adolescent growth may coincide with pregnancy, leading to maternal-fetal competition for nutrients, increasing the risk of low birth weight and neonatal mortality (19).

Bangladesh is one of the most vulnerable countries in the South Asian region concerning child marriage, which could relate to adolescent pregnancy (20); thereby, it is crucial to investigate whether adolescent pregnancy could affect infant mortality. While previous global studies have primarily focused on factors such as antenatal care, birth size, wealth index, maternal education, occupation, and domestic violencee (21–23), as well as modifiable risk factors for neonatal mortality (24), the role of early pregnancy remains underexplored, particularly in Bangladesh. Notably, Noori N, et al. (25) analyzed DHS data from 46 LMICs in sub-Saharan Africa and South Asia (2004–2018) and found significantly higher mortality rates among children of adolescent mothers compared to those of mothers aged 23–25, even after adjusting for covariates (e.g., rural/urban residence and maternal education), with maternal health-seeking behavior potentially mediating this relationship. This study aimed to explore explanatory factors for the association between adolescent pregnancy and infant mortality, which were adjusted for across three levels: sociodemographic factors, contextual factors, and healthcare utilization factors.

## Material and methods

### Study design and data source

This cross-sectional study is based on data from the Bangladesh Demographic and Health Surveys (BDHS) 2017–18. The survey employed a cluster sampling method to collect data from both urban and rural areas, covering all eight divisions of the country. The BDHS 2017–18 surveyed 20,127 ever-married women aged 15–49 years, from which a subset of 8,759 children with available data on whether the child was alive or had died within 12 months of birth, as well as information on maternal age at first birth. This approach ensured that both the predictor (maternal age) and the outcome (infant mortality) pertained to the same child in each case. Exclusions were made for stillbirths (*n* = 222) and multiple births (e.g., twins and triplets), as these cases present unique and complex health conditions warranting separate investigation, consistent with prior research (7).

In the BDHS survey, socio-demographic factors such as maternal education and household income were measured at the time of the survey, not during pregnancy or childbirth. However, birth history data were collected retrospectively, with mothers reporting all births, including birth dates, survivorship status, and either the current age or age at death for each child.

To improve the accuracy of age reporting in Bangladesh, strategies such as cross-checking with newborn birth cards, and verifying the mother's age using formal documents (e.g., birth certificates, ID cards) were implemented. Data collectors also used comparisons with significant historical events or other family members' ages to reduce discrepancies.

The DHS emphasizes ensuring an adequate sample size for data quality and recommends using sample weights to account for disproportionate sampling and non-response. These weights ensure that the data remains representative of the national population. It is important to note that all data were self-reported, with no medical records reviewed.

## Variables measurement

### Dependent variable

The dependent variable in this study is the infant mortality status, which has been categorized into two groups. If infants alive or dead within 12 months of life, they were categorized into alive = 1 and dead = 0.

### Independent variable

The independent variable in this study was the *mother's age at first birth*, which was classified into four groups, recoded as: <16-year = 2, 16–17 years = 2, and 18–19 years = 3, >19-year = 4. More than 19 years was selected as the reference group for comparison with the other groups.

### Potential covariates

The covariates were chosen based on a combination of existing factors widely recognized in the literature as significant influences on maternal and infant health outcomes (4, 5, 7, 8, 10–16), as well as the availability of data within the BDHS dataset. Pearson (*r*) correlations were used to assess the relationships among the independent variables to identify multicollinearity. No signs of multicollinearity (*r* > 0.7) were found among the independent variables.

The variables were classified into socio-demographic, contextual, and healthcare utilization factors.

Socio-demographic factors—*Father's occupation* is categorized into three groups: not working = 0, service holder = 1, and agriculture = 2. *Wealth status* was categorized into five groups, called quintiles:: poorest = 0, poorer = 1, Middle = 2, richer = 3 and richest = 4. These quintiles are estimated using data on household assets, such as housing conditions, water access, and ownership of goods. The households are then ranked from lowest to highest wealth and divided into five equal parts, representing the wealth distribution across the population. Given that most of the population in Bangladesh is Muslim, the variable *Religion* was categorized into two groups: Islam = 1 and Others = 2.

Health status, as indicated by *Body Mass Index (BMI)* of mothers was considered a continuous scale in this study. The BMI is determined by dividing a person's weight in kilograms by the square of their height in meters (kg/m^2^). For the BDHS 2017–18 survey, the BMI is calculated for women aged 15–49 who are not pregnant and have not given birth in the two months before the survey. The BMI values are classified into four groups based on predetermined cut-off points: underweight ≤ 18.5, healthy weight = 18.5–24.9, overweight = 25.0–29.9, and obesity ≥ 30. Contextual factors—*Place of residence* was categorized into two groups: rural = 1 and urban = 2. *Place of delivery* was recategorized into two groups: delivered at home = 1 and delivered at institution = 2. *Knowledge of family planning (FP)* was divided into two groups: mothers who had knowledge of FP were categorized as 1 and those who had no knowledge of FP were categorized as 0. The mother's knowledge of FP was assessed based on several questions related to her access to mass media exposure, such as *reading newspapers*, *watching television*, *owning a mobile phone*, and *listening to the radio*. All these mass media exposures were categorized into yes = 1 or no = 0. Moreover, the variable *attended FP session* was categorized into yes = 1 (any session) or no = 0 (no session).

Healthcare utilization factors—*receiving ANC and PNC* considered binary variables and categorized them as care received = 1 and no care received = 0, respectively. Mothers who received at least one ANC and PNC counted as “care received” in this study.

## Data analysis

All statistical analyses were conducted using Stata version 15.0. A sampling weight was considered for all analyses to normalize the data at a national level and to minimize the standard errors. To create the sampling weight, a complex sample plan was developed by aggregating three variables: (i) primary sampling units, (ii) sample strata, comprising a representative sample, and (iii) sample weights, which calculate the sampling probability for each stage.

A descriptive analysis was used to summarize the basic characteristics of the study population. Categorical variables were presented using frequency and percentages, while continuous variables were presented using means and standard deviation. A Pearson Chi-square test was employed to evaluate the statistical significance of the observed differences between categorical variables, including maternal age at first birth, and the dependent variable. Additionally, a *t*-test was utilized for all continuous covariates to assess the significance of the differences between these covariates and the dependent variable.

Prior to executing logistic regression models, variance inflation factors (VIFs) were estimated to check for multicollinearity between explanatory variables, with a VIF value greater than 10 assumed to indicate high collinearity. No VIF value greater than 0.5 was found, indicating no collinearity between the independent variables (26). Only covariates with a *p*-value less than 0.25 from the Chi-square and *t*-test were entered into the univariate logistic regression (27). A three-step process was followed in the logistic regression model. In model 1, a univariate logistic regression was performed to see an association of each independent variable (including covariates) with the outcome. In Model 2, a multivariate analysis was conducted, incorporating sociodemographic factors (i.e., fathers' occupation and religion) and contextual factors (i.e., place of delivery, television viewing, mobile phone ownership, and knowledge of family planning) along with mothers' age at first birth. Additionally, in Model 3, all the variables from Model 2, including sociodemographic and contextual factors, were combined with healthcare-related variables to assess their impact comprehensively. For all models, odds ratios (OR) with 95% confidence intervals (CI) were calculated to identify the relationship between each independent variable and infant mortality. A two-tailed test was used, and the level of significance for all analyses was set at *p* ≤ 0.05.

## Ethical approval

The BDHS is publicly available for performing research, reviewed and approved by the ICF Macro Institutional Review Board (USA), and also by the National Research Ethics Committee of the Bangladesh Medical Research Council (Dhaka, Bangladesh). During data collection, informed consent from each participant was obtained ensuring confidentiality. Ethical approval for using the data was obtained from ICF International Rockville, Maryland, USA, in October 2022. The analyses performed in this study were carried out following relevant guidelines and regulations.

## Results

The mean age of the mothers at first birth was 18.53 years. Among mothers with higher education, 9.67% experienced infant loss, compared to 45.12% and 36.28% for mothers with intermediate and elementary education, respectively. Of all, 25.3% of the women who experienced infant loss came from the poorest families, compared to only 16.16% from the wealthiest family background. Moreover, 291 (3.6%) out of 7,989 Muslim mothers reported infant deaths, compared to 37 (5.0%) out of 704 non-Muslim mothers. Additionally, 46.67% of mothers who reported infant deaths gave birth at home, compared to 53.33% in some institutions (Table 1). However, the study identified the following variables to be included in the Binary logistic regression analyses: the mother's education, father's age, religion, delivery place, watching television, owning a mobile phone, and receiving antenatal care (ANC) or postnatal care (PNC).

**Table 1:** Descriptive characteristics and association of infant death status from first birth with mother's age group and other independent variables (*n* = 8,759).

Factors	Infant status			
	Alive	Dead	Missing	*p*-value
Age of mothers at first birth				
<16	1,217 (14.48%)	66 (20.12%)		0.074
16–17	1,078 (12.83%)	43 (13.11%)		
18–19	3,580 (42.61%)	125 (38.11%)		
>19	2,527 (30.08%)	94 (28.66%)		
Missing			29	
Father's occupation				
Not working	230 (2.70%)	11 (3.40%)		0.780
Service holder	6,568 (78.10%)	253 (77.10%)		
Agriculture	1,616 (19.20%)	64 (19.50%)		
Missing			328	
Wealth status^a^				
Poorest	1,835 (21.84%)	83 (25.30%)		0.448
Poorer	1,684 (20.04%)	65 (19.82%)		
Middle	1,497 (17.82%)	62 (18.90%)		
Richer	1,667 (19.84%)	65 (19.82%)		
Richest	1,719 (20.46%)	53 (16.16%)		
Missing			29	
Religion				
Islam	7,698 (91.62%)	291 (88.72%)		0.047
Other	704 (8.38%)	37 (11.28%)		
Missing			29	
BMI				
Mean (*n*)	22.66 (8,268)	22.57 (326)	165	0.687
Place of residence				
Urban	2,930 (34.87%)	119 (36.28%)		0.619
Rural	5,472 (65.13%)	209 (63.72%)		
Missing			29	
Place of delivery				
Home	2,561 (50.05%)	84 (46.67%)		<0.001
Institution	2,556 (49.95%)	96 (53.33%)		
Missing			3,462	
Read newspaper				
No	7,485 (89.09%)	295 (89.94%)		0.701
Yes	917 (10.91%)	33 (10.06%)		
Missing			29	
Watch television				
No	3,199 (38.07%)	140 (42.68%)		0.042
Yes	5,203 (61.93%)	188 (57.32%)		
Missing			29	
Own mobile phone				
No	3,159 (37.60%)	135 (41.16%)		0.215
Yes	5,243 (62.40%)	193 (58.84%)		
Missing			29	
Listen to radio				
No	7,952 (94.64%)	312 (95.12%)		0.701
Yes	450 (5.36%)	16 (4.88%)		
Missing			29	
Knowledge of FP				
No	6,990 (83.19%)	284 (86.59%)		0.077
Yes	1,411 (16.79%)	44 (13.41%)		
Missing			30	
Attended FP session (any)				
No	8,344 (99.31%)	326 (99.39%)		0.992
Yes	57 (0.68%)	2 (0.61%)		
Missing			30	
Received ANC (any)				
No	399 (4.75%)	7 (2.13%)		0.077
Yes	4,500 (53.56%)	100 (30.49%)		
Missing			3,753	
Received PNC (any)				
No	1,659 (19.75%)	56 (17.07%)		<0.001
Yes	3,234 (38.49%)	51 (15.55%)		
Missing			3,759	

^a^ Wealth status determined by ranking households based on assets like housing, water access, and goods ownership, then dividing them into five equal groups, called quintiles, representing different levels of wealth.

FP, family planning; ANC, antenatal care, PNC, postnatal care.

Both groups of mothers—those who reported infant death and those who had live children at their first birth—did not significantly differ in terms of the mother's age, father's occupation, wealth index, BMI, place of residence, knowledge of FP, and mothers who received any ANC (Table 1).

Table 2 shows the results from logistic regression of factors associated with infant mortality. Infants born to mothers under 16 years had higher odds of mortality compared to infants born to mothers over 19 years, and the results were statistically significant in model 1 (crude model), (OR: 1.45, 95% CI: 1.05, 2.01) and persisted in Model 2 (OR: 1.41, 95% CI: 1.01–1.96) after adjusting for sociodemographic factors such as maternal age, paternal occupation, and religion, as well as contextual factors like place of delivery, television access, mobile phone ownership, and knowledge of family planning. However, after further adjusting for healthcare utilization factors, including antenatal and postnatal care, in Model 3, the association was no longer statistically significant (AOR: 1.06, 95% CI: 0.56–1.99).

**Table 2:** Results from a binary and multivariate logistic regression model exploring the association between infant death status, maternal age group, and other independent variables.

Dependent variable (Alive = 0, Death = 1)	Model 1 (*n* = 8,759)		Model 2 (*n* = 5,304)		Model 3 (*n* = 5,003)	
	OR (95% CI)	*p*-value	OR (95% CI)	*p*-value	OR (95% CI)	*p*-value
Mother's age at first birth						
<16	1.45 (1.05–2.01)	0.022	1.41 (1.01–1.96)	0.044	1.06 (0.56–1.99)	0.863
16–17	1.07 (0.74–1.55)	0.710	1.02 (0.70–1.48)	0.935	0.69 (0.34–1.42)	0.318
17–19	0.93 (0.71–1.23)	0.649	0.91 (0.69–1.20)	0.503	0.76 (0.47–1.23)	0.265
>19	1		1		1	
Father's occupation						
Not working	1		1		1	
Service holder	0.81 (0.43–1.49)	0.492	0.81 (0.44–1.51)	0.507	0.74 (0.56–1.68)	0.412
Agriculture	0.83 (0.43–1.60)	0.572	0.79 (0.41–1.53)	0.488	0.73 (0.51–1.73)	0.425
Religion						
Islam	1		1		1	
Other	1.39 (0.97–1.97)	0.066	1.42 (0.99–2.03)	0.058	2.36 (1.39–4.01)	0.001**
Place of delivery						
Home	1		1		1	
Institution	1.15 (0.85–1.54)	0.373	0.85 (0.66–1.09)	0.193	0.83 (0.50–1.38)	0.471
Watch television						
No	1		1		1	
Yes	0.83 (0.66–1.03)	0.092	0.85 (0.66–1.09)	0.193	1.12 (0.72–1.74)	0.612
Own mobile phone						
No	1		1		1	
Yes	0.86 (0.69–1.08)	0.192	0.90 (0.72–1.14)	0.392	1.01 (0.66–1.52)	0.977
Knowledge of FP						
No	1		1		1	
Yes	0.77 (0.56–1.06)	0.108	0.83 (0.59–1.16)	0.273	0.99 (0.56–1.76)	0.992
Received ANC (any)						
No	1				1	
Yes	1.27 (0.58–2.74)	0.549			1.33 (0.59–2.99)	0.497
Received PNC (any)						
No	1				1	
Yes	0.47 (0.32–0.69)	<0.001			0.42 (0.26–0.67)	<0.001

**Significance in *p*-value ≤ 0.01.

Model 1: Crude analysis.

Model 2: Adjusted for sociodemographic factors (i.e., mothers' age, fathers' occupation, and religion) and contextual factors (i.e., place of delivery, watch television, own mobile phone, and knowledge on FP).

Model 3: Adjusted for all the variables in Model 2 (i.e., sociodemographic factors and contextual factors) and additionally all variables in healthcare utilization (i.e., received ANC and PNC).

FP, family planning; ANC, antenatal care; PNC, postnatal care.

If the mother attended at least one ANC session was recorded “yes” here; If the mother attended at least one PNC session was recorded “yes” here.

## Discussion

The present study reveals a significant increase in infant mortality among mothers under 16 years old compared to those aged 19 years or older. This finding was robust across demographic and contextual factors but became non-significant after adjusting for healthcare utilization. Additionally, the study identifies religion and PNC as significantly associated with infant mortality.

While this study does not negate the association between adolescent pregnancy and infant mortality, recent evidence from a study across 46 Low- and Middle-Income Countries (LMCs) in Sub-Saharan Africa and South Asia highlights adolescent pregnancy as a recognized health risk for both mother and child (25). Unlike Noori et al. (25), which offers a broader regional perspective (including Bangladesh), our study provides a more in-depth exploration of Bangladesh, a country facing distinct health challenges and disparities. A key contribution of our research is the finding that, once healthcare utilization factors, such as antenatal and postnatal care, are accounted for, the previously observed relationship between adolescent pregnancy and infant mortality loses its statistical significance. This underscores the crucial role of healthcare access in mitigating infant mortality and provides a more nuanced understanding of adolescent pregnancy's impact within Bangladesh. Furthermore, our study offers a more granular analysis by thoroughly adjusting for a comprehensive set of covariates, including sociodemographic, contextual, and healthcare utilization factors. The study also notes a decline in absolute mortality rates over time but underscores that the age gradient persists across different time periods. It recommends further investigation into biological and social factors that mediate the relationship between adolescent pregnancy and child mortality.

Similarly, the 2011 BDHS study found that Bangladesh's infant and child mortality rates are higher among rural populations and families with lower socio-economic status (28). Also, the study underlined that access to healthcare services and maternal education were essential factors in reducing infant and child mortality in Bangladesh. Therefore, this study argues that infant mortality can be attributed to several factors other than the mother's age alone.

The study also found that religion was significantly associated with infant mortality. Respondents of other religions had higher odds of infant death compared to Muslims in the present study. The possible reasons for this finding could be differences in religious beliefs and practices that may have influenced maternal and child health-seeking behavior. Religious differences significantly influence maternal and child health-seeking behaviors. Some religious communities may favor traditional healing over biomedical care, causing delays in seeking necessary healthcare for mothers and infants (29). Additionally, specific religious doctrines may discourage certain medical procedures, particularly in family planning and childbirth (30). Literature indicates that various religious affiliations can introduce unique barriers to healthcare access, including socioeconomic disparities. Religious minority groups often experience higher poverty levels, limiting their healthcare access (31). It highlights the importance of understanding cultural and religious factors that have affected maternal and child health outcomes and the need to tailor interventions to address these factors. However, a significant shift can be observed in this study compared to similar studies. For example, using data from MICS 2019, a study found that non-Muslim women in rural areas had a 7.5% lower risk of offspring mortality than their Muslim counterparts (14). Similarly, other findings showed that Muslim children are at an increased risk of neonatal death compared to non-Muslims due to accessibility to adequate maternal care (15). Additionally, using the same dataset BDHS 2017–18, Muslim respondents had a lower likelihood of delivering a child in an institution than non-Muslim religions, associated with an increased risk of infant mortality (12). Nevertheless, using the BDHS data from 2017 to 18, a study also reported that religion has a significant relationship with infant mortality. The researchers found that the infant mortality rate was higher among non-Muslim infants compared to Muslim infants (5). So, further research is needed to understand better the role of religion in infant mortality in Bangladesh to develop targeted interventions that address the specific needs of different religious groups.

Finally, this study found that receiving PNC was significantly associated with lower odds of infant mortality. It is consistent with previous studies that have shown the importance of PNC in improving maternal and child health outcomes (32–34). PNC provides an opportunity to identify and manage maternal and infant health problems, including monitoring the infant's growth and development. Therefore, this study emphasizes the need for improving access to PNC for all mothers in Bangladesh, particularly those living in rural and slum areas where access to health facilities is limited (35).

## Strengths and limitations

Several strengths of this study should be highlighted. Firstly, the study used a large and nationally representative sample of women in Bangladesh, providing a comprehensive overview of the country's maternal and child health outcomes. Using such a sample increases the generalizability of the results to the larger population of women in the country. The study examined the relationship between maternal age and infant mortality while controlling for other relevant factors, including socioeconomic and healthcare utilization variables. To minimize potential sources of bias and uncertainty, the study design included steps such as anonymizing the survey and assuring participants that their responses would be kept confidential.

The study has some limitations that need to be considered when interpreting its results. The study relied on self-reported data, which may be subject to recall and social desirability biases, meaning that some participants may not accurately remember or report certain information. Although data confidentiality measures were in place, recall bias may still affect the accuracy of the reported information. The study would have been further strengthened by additional analysis of effect modification, but limitations in the questionnaire's design did not allow for this. For example, in the questionnaire, if a mother received at least one PNC visit before the interview, it was coded as “received PNC ever,” but it was not specified whether this PNC was received for her first pregnancy or subsequent pregnancies.

The study's cross-sectional nature means it is impossible to establish a causal relationship between maternal age and infant mortality. We acknowledge that the timing of certain covariates, such as television watching, remains an issue. Like most other variables measured at the time of the survey, it may not accurately reflect the context surrounding each individual case of adolescent pregnancy and infant mortality. This is particularly relevant for women whose infants have died, as this occurred before the survey, possibly up to a year earlier. Additionally, we consider that infant mortality may have influenced the covariates we included. We thus emphasize the potential for reverse causality and the challenges of establishing causal links in cross-sectional studies. Moreover, there are limitations due to recall bias in data collection birth history, such as selective omission of non-surviving births, leading to underestimation of child mortality. Other issues include birth date displacement, misreporting age at death, and assumptions that maternal reports of child mortality are accurate, despite potential correlations between maternal and child mortality risks.

One key limitation of our study is the possibility of residual confounding. Although we adjusted for various covariates such as sociodemographic, health status, contextual, and healthcare utilization factors, there may be other important covariates or confounders that we could not account for, such as detailed nutritional information, household environmental factors, or psychosocial stressors, because they were not available in the dataset. These unmeasured factors could potentially influence infant mortality (7). Additionally, self-reported recall data on variables such as birth history and antenatal care may be subject to recall bias, affecting the accuracy of our findings.

While our adjustments improve the robustness of the results, the loss of statistical significance in Model 3, after the additional adjustment for healthcare utilization factors along with sociodemographic and contextual factors, suggests that healthcare utilization factors may play a larger role in mediating the relationship between maternal age and infant mortality than initially anticipated. Nevertheless, unmeasured covariates could still affect this complex relationship, and future studies with more comprehensive data are needed to explore these influences more thoroughly. The dataset we analyzed for this study included only ever-married women, excluding unmarried adolescent mothers, who might face unique healthcare, financial, and social challenges linked to higher infant mortality. Future research should include this group for a more comprehensive understanding of these factors. Another limitation is the potential for response bias, particularly on sensitive topics such as reproductive health, which may impact data quality. Additionally, the BDHS data is based on a population sample, which may not fully represent the entire population due to sampling error. Data quality may also be affected by incomplete or inaccurate data collection, missing data, and errors in data entry. Some data may not have been available or collected, limiting the comprehensiveness of the information. The sampling methodology used may introduce selection bias, which could affect the results. For example, the survey only included a limited number of questions related to infant death and the mother's age, which may not provide a complete understanding of adolescent motherhood and its complexity. Future studies may require a more comprehensive examination of adolescent pregnancy and its relationship to infant mortality. Furthermore, we recognize that WHO guidelines recommend at least eight ANC visits to achieve optimal maternal and child health outcomes (36). While we based our analysis on the available data, which used at least one ANC visit as the minimum threshold, we acknowledge that this approach is limited. This underscores the importance of obtaining more detailed ANC visit data in future studies.

## Conclusions

The findings suggest that delaying the first birth from adolescence to adulthood may reduce infant mortality. This relationship may not be influenced by sociodemographic factors, but it seems to be influenced by healthcare utilization factors. After controlling healthcare utilization factors, maternal age did not matter for infant mortality, rather healthcare utilization factor—perceived any PNC was found to be a protective factor for infant mortality. Further, the results suggest that religion plays an important role in terms of infant mortality. For policymakers, the study findings suggest that efforts to improve maternal and child health should prioritize promoting adolescent reproductive health and family planning education, especially for young girls. It also highlights the need to improve access to quality healthcare services for mothers and newborns, including postnatal care services. Targeted interventions are also necessary to achieve the SDG's target of reducing child mortality, such as improving maternal health outcomes, reducing religious barriers, and reducing disparities in maternal and child health.

## Data Availability

Publicly available datasets were analyzed in this study. This data can be found here: The DHS analyzed in this study is publicly available on the DHS Program's page at https://dhsprogram.com/.
